# Short Linear Motifs Orchestrate Functioning of Human Proteins during Embryonic Development, Redox Regulation, and Cancer

**DOI:** 10.3390/metabo12050464

**Published:** 2022-05-21

**Authors:** Susanna S. Sologova, Sergey P. Zavadskiy, Innokenty M. Mokhosoev, Nurbubu T. Moldogazieva

**Affiliations:** 1 Nelyubin Institute of Pharmacy, Sechenov First Moscow State Medical University, (Sechenov University), 119991 Moscow, Russia; sologova_s_s@staff.sechenov.ru (S.S.S.); zavadskiy_s_p@staff.sechenov.ru (S.P.Z.); 2 Department of Biochemistry and Molecular Biology, Pirogov Russian National Research Medical University, 117997 Moscow, Russia; imokhosoev@mail.ru

**Keywords:** short linear motifs, alpha-fetoprotein, cancer, redox regulation, embryonic development, bioinformatics

## Abstract

Short linear motifs (SLiMs) are evolutionarily conserved functional modules of proteins that represent amino acid stretches composed of 3 to 10 residues. The biological activities of two short peptide segments of human alpha-fetoprotein (AFP), a major embryo-specific and cancer-related protein, have been confirmed experimentally. This is a heptapeptide segment LDSYQCT in domain I designated as AFP_14–20_ and a nonapeptide segment EMTPVNPGV in domain III designated as GIP-9. In our work, we searched the UniprotKB database for human proteins that contain SLiMs with sequence similarity to the both segments of human AFP and undertook gene ontology (GO)-based functional categorization of retrieved proteins. Gene set enrichment analysis included GO terms for biological process, molecular function, metabolic pathway, KEGG pathway, and protein–protein interaction (PPI) categories. We identified the SLiMs of interest in a variety of non-homologous proteins involved in multiple cellular processes underlying embryonic development, cancer progression, and, unexpectedly, the regulation of redox homeostasis. These included transcription factors, cell adhesion proteins, ubiquitin-activating and conjugating enzymes, cell signaling proteins, and oxidoreductase enzymes. They function by regulating cell proliferation and differentiation, cell cycle, DNA replication/repair/recombination, metabolism, immune/inflammatory response, and apoptosis. In addition to the retrieved genes, new interacting genes were identified. Our data support the hypothesis that conserved SLiMs are incorporated into non-homologous proteins to serve as functional blocks for their orchestrated functioning.

## 1. Introduction

Proteins are key cellular components involved in practically all the essential processes in a living organism. The functioning of proteins is assured by the presence of functionally important regions and modules, which can be organized at different structural levels, from primary through secondary to tertiary, three-dimensional (3D) structures [1]. These modules include independently folded 3D domains, secondary structure elements (SSEs), and short linear motifs (SLiMs), which provide multimodular and multifunctional features of many proteins [2,3].

Functional modules of proteins have been attained over a long evolutionary time and are implicated in a vast array of biological processes, including metabolism, cell division, stress response, signal transduction, and cell-to-cell and cell-to extracellular matrix (ECM) interactions [4]. SLiMs are short amino acid stretches composed of 3 to 10 highly conserved residues that can be involved in protein–protein interactions (PPIs) underlying various protein functions [5]. Such motifs have been implicated in many fundamental processes, including Arg-Gly-Asp (RGD) tripeptide, which provides an interaction of ECM proteins with their receptors, integrins [6]. Other examples are the KDEL motif, which marks proteins for their retainment in the endoplasmic reticulum (ER) [7], and Src-homology 3 (SH3)-binding proline-rich motif, PxxP [8].

SLiMs are stable in a variety of proteins and protein families, but the same sequence can code for different native structures that are covered by current fragment libraries [9]. They can be intertwined and overlapped and are incorporated into proteins to serve as functional building blocks. This multiplicity of structural states for a single sequence explains why the same structure may be found in a variety of unrelated non-homologous proteins. Thus, despite highly differentiated amino acid sequences and 3D structures, proteins may share similar functions. These protein segments can have a native conformation around the local minimum of potential energy function, allowing proteins to reuse the same patterns of recurring motifs [10]. This reuse enables transition from one conformation to another by sampling conformational assemblies of the protein backbone.

Domain–SLiM interactions mediate many PPI pathways, whereas post-translational modifications of a SLiM may provide a switch from its interaction with one domain to another [11,12]. Most domains maintain unique amino acid conservation patterns, which suggest that they can bind SLiMs with high intrinsic specificity, and this influences the PPIs [13]. Moreover, SLiMs have evolved to coadapt their specificity and affinity to the functional diversity of domain–peptide interactions [14], and the interplay between a modular domain, such as SH3, and its host protein is important in establishing the specificity to wire PPI networks [15]. However, their very small size and poorly folded nature make SLiMs difficult to detect experimentally. Many computational and bioinformatics tools have been developed for PPI analysis on the basis of known SLiM-recognition domains [16].

Here, we used a local sequence alignment algorithm for a computational search of SLiMs that have sequence similarity to two biologically active peptide segments of human alpha-fetoprotein (AFP), a major mammalian embryo-specific and cancer-related protein [17]. One of these segments, LDSYQCT, encompasses amino acid residues from 14 to 20 in mature AFP and is designated as AFP_14–20_ [18]. Another one is a *C*-terminal fragment of AFP-derived growth inhibitory peptide (GIP) composed of nine residues, EMTPVNPGV, and designated as GIP-9 [19]. It has been experimentally shown that the GIP peptide displays the ability to inhibit mouse uterine cell proliferation and anticancer effects in MSF-7 breast cancer cells [20]. AFP_14–20_ demonstrated an immunomodulatory capability in a phytohemagglutinin (PHA)-activated lymphocyte culture [21]. We used gene ontology (GO) functional enrichment, Kyoto encyclopedia of genes and genomes (KEGG) pathway, metabolic pathway, and PPI network analyses to categorize human proteins containing both AFP_14–20_-like and GIP-9-like motifs. We found that the retrieved proteins belong to transcription factors, cell adhesion proteins, cell signaling proteins, ubiquitin-activating and conjugating enzymes, and oxidoreductase enzymes and are involved in embryonic development, cancer progression, and redox regulation.

## 2. Results

### 2.1. Human Proteins Aligned to Human AFP Segments

In total, 222 human proteins with sequence similarity to the AFP_14–20_ segment and 55 proteins with sequence similarity to the GIP-9 segment of human AFP were retrieved from UniprotKB knowledgebase. These proteins include AFP itself, putative proteins, and uncharacterized proteins from both Swiss-Prot and TrEMBL sections.

Table 1 contains representative proteins aligned to the AFP_14–20_ segment; the lower the E-value, the more significant the sequence alignment. As shown in Table 1, proteins significantly aligned with the AFP_14–20_ segment are involved in a diversity of functions, including cell proliferation and differentiation, development, metabolism, immune/inflammatory response, redox homeostasis, and apoptosis. These proteins include transcription factors, such as tripartite motif (TRIM)-containing protein 3, haematopoietically-expressed homeobox protein HHEX, and zinc finger protein 714. The AFP_14–20_ segment was also found in proteins that are involved in DNA replication, cell cycle regulation, and cell division, including DNA polymerases, nucleotide transferases, and growth factors. Multiple epidermal growth factor (EGF)-like repeat-containing, calcium-binding, and membrane-bound extracellular matrix proteins, such as members of neurogenic locus notch, von Willebrand factor A domain-containing protein and fibulin-4, which are crucial for cellular homeostasis and functioning, were also identified. Additionally, ubiquitin-activating enzyme (E1) and ubiquitin-protein ligase (transferase) enzyme (E3), which are involved in protein modification and protein quality control, were also aligned to the AFP_14–20_ segment. Interestingly, there were oxidoreductases involved in oxidative stress response among the retrieved proteins, including prostaglandin G/H synthase 1, glutathione S-transferase LANCL1, and prolyl hydroxylase.

Table 2 shows the most representative proteins that were aligned with high significance to the GIP-9 segment of human AFP. They include developmental proteins, such as isoforms of C-C motif chemokine 4-like; and Wnt-signaling regulators, such as AXIN2 and L1 cell adhesion molecule (L1CAM or CD171). Like AFP_14–20_, the GIP-9 segment was aligned to various proteins with transcription factor activity, such as homeobox protein Hox-C5 (HOXC5), forkhead box protein O1 (FOXO1), and zinc finger proteins 547 and 213. Among the aligned proteins, there were cell-cycle regulators, such as antiproliferation factor 3 (BTG3)-associated nuclear protein and cyclin-dependent kinase inhibitor 1B. There were also proteins involved in DNA replication, repair, and recombination. Additionally, various proteins with receptor activity, including IGF-like family receptor 1, brain-specific angiogenesis inhibitor (BAI) family proteins, and E3 ubiquitin-protein ligase TRIM35, were aligned to GIP-9. Growth hormone receptor (GHR) was also involved in metabolic regulation. Importantly, various oxidoreductase enzymes, such as ceruloplasmin and pyridoxine 5’-phosphate oxidase (PNPO), were also aligned to the GIP-9 segment.

### 2.2. Biological Process Categories

**Figure 1:**
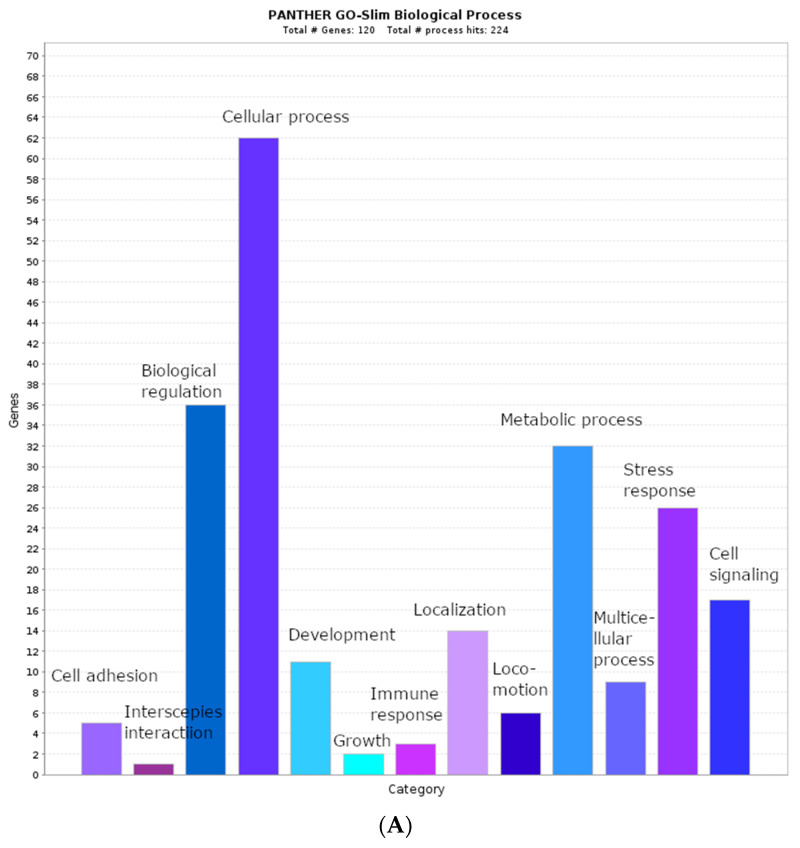

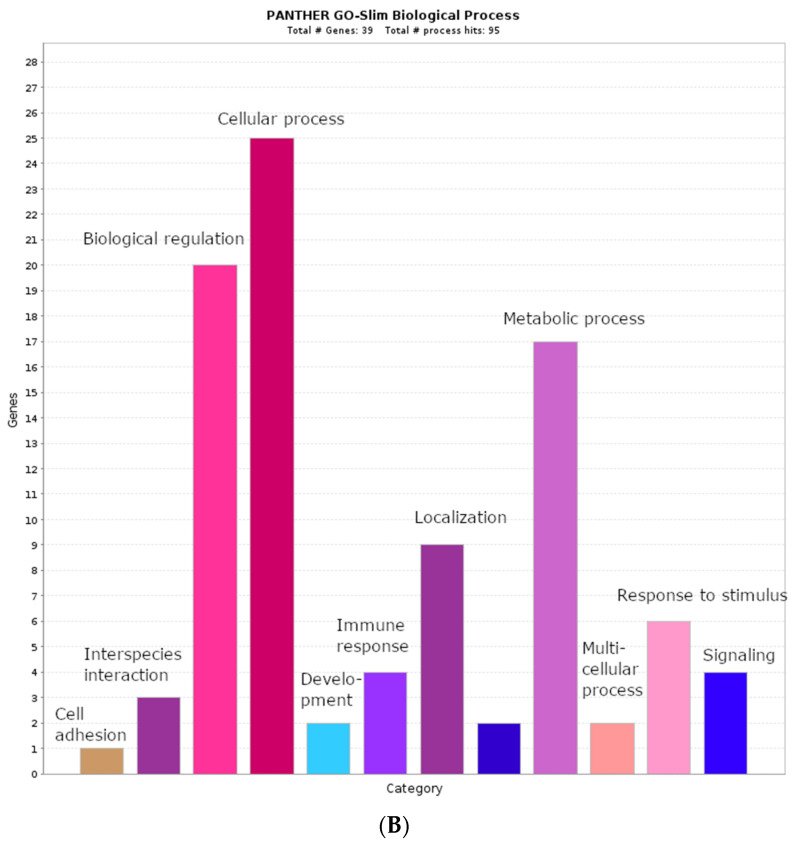
Representation of gene-ontology-based biological process enrichment categorization of genes encoding (**A**) AFP_14–20_-like motif-containing proteins and (**B**) GIP-9-like motif-containing proteins. The gene list enrichment analysis tool of PANTHER17.0 was applied. All query genes were retrieved from UniprotKB knowledgebase and then converted to ENSEMBL gene IDs.

We performed GO term categorization to assess the involvement of genes from our gene list in various biological processes. This enabled the identification of unique human genes and total gene amounts associated with a given GO biological process term. In total, we identified 120 and 39 unique genes encoding proteins that contain AFP_14–20_-like and GIP-9-like motifs, respectively. The results of biological process enrichment of the genes encoding AFP_14–20_-like motif-containing proteins are shown in Figure 1A. With the PANTHER17.0 classification system, 116 of 120 unique genes were mapped to the whole human genome, and a total of 224 biological process hits associated with our gene list were found. The most statistically significant GO categories (*p*-value ˂ 0.05) belonged to developmental processes, such as “multicellular organism development”, “tissue development”, “biological adhesion”, and “positive regulation of cell differentiation”. At a *p*-value cutoff of 0.2, more biological process terms were identified: 36 genes were implicated in biological regulation, 32 genes were implicated in metabolic processes, 26 genes were implicated in response to various stress stimuli, and 17 genes were implicated in cell signaling. These GO categories can overlap with one another. For example, 62 genes were involved in variety of cellular processes, including protein biosynthesis, protein transportation, protein quality control, metabolism, cellular component organization, cell communication, signal transduction, and cellular response to chemical stimulus.

Categorization of genes encoding GIP-9-like motif-containing proteins on the basis of GO biological process terms is shown in Figure 1B. Of the 39 unique genes, 37 were mapped to the whole human genome and were involved in 95 biological processes. The overrepresented genes were involved in biological regulation (20), metabolic processes (17), localization (9), response to stimulus (6), cell signaling (4), and immune response (4). Due to overlapping of various biological process categories, a general term, “cellular process”, included 25 genes subcategorized as transcriptional regulation, cytokine signaling, ubiquitin-mediated protein degradation, DNA replication/repair, cytoskeletal organization, ion channel regulation, protein transport, and localization.

### 2.3. Molecular Function Categories

**Figure 2:**
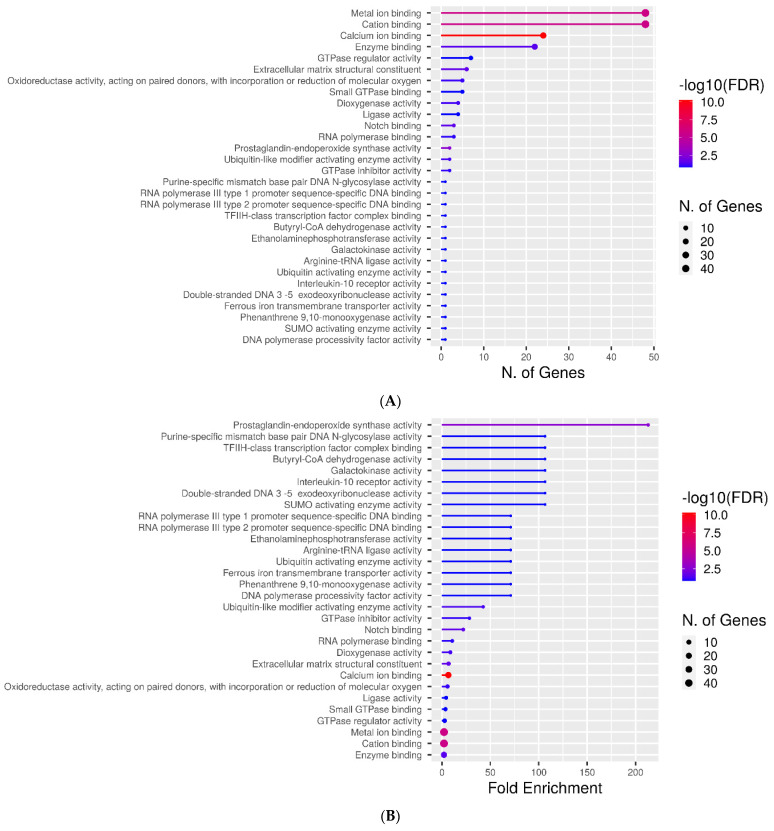

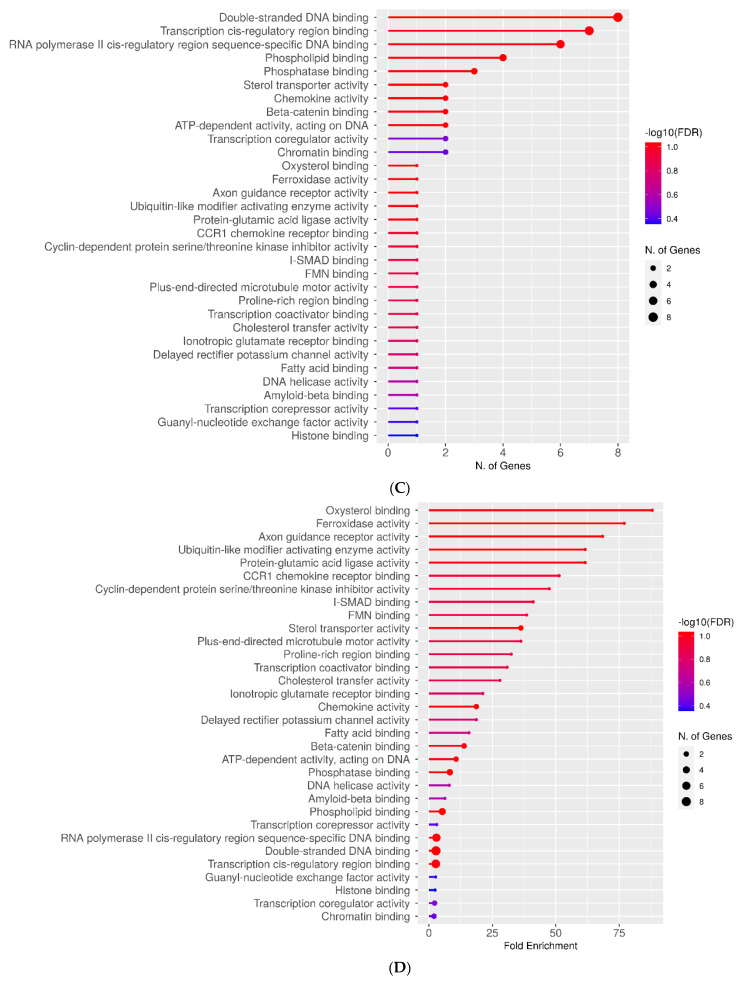
Gene ontology term-based molecular function categorization of genes encoding (**A**,**B**) AFP_14–20_-like motif-containing proteins and (**C**,**D**) GIP-9-like motif-containing proteins with the use of the ShinyGO v075 suite. Categories are ranked by (**A**,**C**) number of genes and (**C**,**D**) fold enrichment. Lolipop chats with an aspect ratio of 1.5 and −log10 (FDR) heat maps for each category are shown.

For a more detailed gene set enrichment analysis, molecular function categorization was performed with the use of the ShinyGO v0.75 suite. Figure 2A depicts genes encoding AFP_14–20_-like motif-containing proteins ranked by the number of genes in each category at a *p*-value cutoff of 0.2. As many as 48 genes belonged to the “metal ion binding”, “cation binding”, and “enzyme binding” categories. These molecular function categories are inherent to transcription factors and oxidoreductase enzymes. The additional “calcium ion binding” category is mostly inherent to cell signaling and developmental proteins. Additionally, the “extracellular matrix structural constituent” category evidences the implication of 6 AFP_14–20_-like motif-containing proteins in cell adhesion processes. Figure 2B depicts the ranking of genes of interest by fold enrichment. This shows that oxidoreductase enzymes with prostaglandin-endoperoxide synthase (cyclooxygenase) activity, which are involved in ROS generation and redox regulation, were aligned with the most significance to the AFP_14–20_ segment.

Figure 2C depicts the categorization of genes encoding GIP-9-like motif-containing proteins at a *p*-value cut-off 0.2 by molecular function terms that are ranked by the number of identified genes. Nucleic acid (DNA)-binding activity that is inherent to transcriptional regulators was the most represented term. This was followed by phospholipid and phosphatase binding, sterol transporter, beta-catenin binding, and chemokine and transcription coregulator activities (Figure 2C). However, ranking of these genes by fold enrichment revealed the highest significance of proteins with oxysterol binding and ferroxidase activities that are specific to redox regulation. Axon guidance receptor activity, ubiquitin-activating enzyme activity, CCR1 chemokine receptor binding, and cyclin-dependent serine/threonine kinase inhibitor activity were also inherent to GIP-9-like motif-containing proteins, although with less significance (Figure 2D).

### 2.4. KEGG Pathways

**Figure 3:**
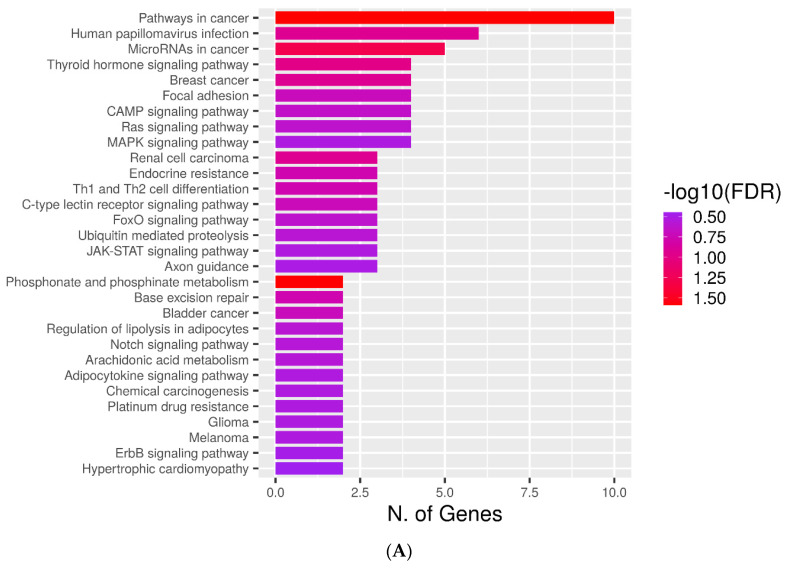

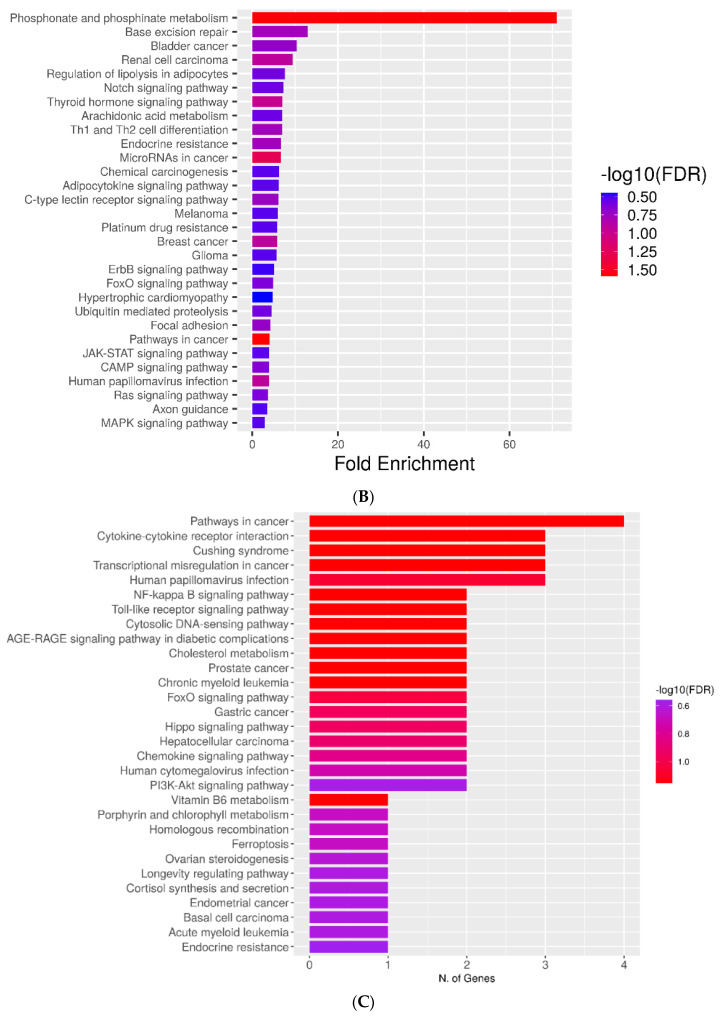

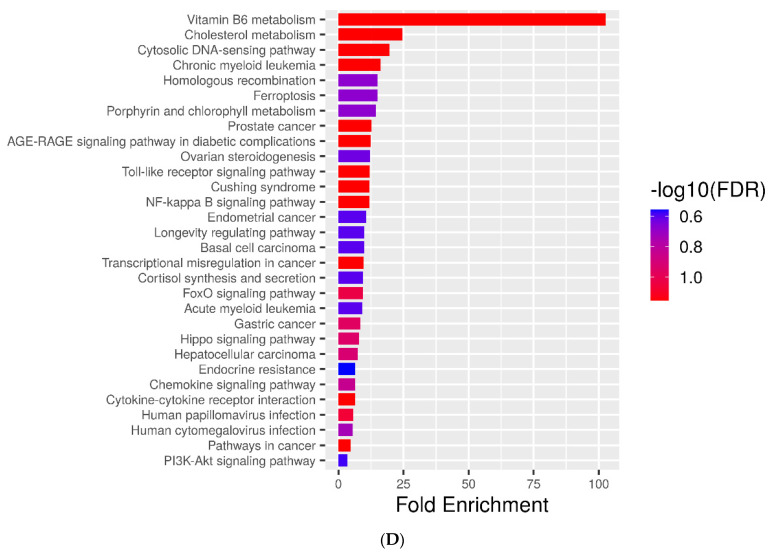
KEGG pathway enrichment analysis of genes encoding (**A**,**C**) AFP_14–20_-like motif-containing proteins and (**B**,**D**) GIP-9-like motif-containing proteins. Rankings by both number of genes (**A**,**B**) and fold enrichment value (**C**,**D**) are shown. Bar plot representation with an aspect ratio of 1.5 and −log10 (FDR) heat maps for each category are shown.

To elucidate molecular mechanisms underlying the functioning of the retrieved proteins, we undertook KEGG pathway enrichment analysis. Figure 3A,C shows that 10 and 4 genes encoding AFP_14–20_-like and GIP-9-like motif-containing proteins, respectively, are involved in cancer-associated pathways. The AFP_4–20_-like motif group includes genes associated with cAMP-signaling, Ras-signaling, MAPK-signaling, FOXO-signaling, JAK-STAT-signaling, notch-signaling, and ErbB-signaling pathways (Figure 3A). Among these pathways are those involved in metabolic processes and drug resistance. The GIP-9-like group includes cytokine signaling, NF-kB-signaling, Toll-like-receptor-signaling, AGE-RAGE-signaling, FoxO-signaling, and PI3K-Akt-signaling pathways (Figure 3C). Therefore, proteins containing the SLiMs of interest mediate the abovementioned signal transduction pathways implicated in cancer initiation and progression.

KEGG pathway ranking by fold enrichment showed the involvement of genes encoding AFP_14–20_-like motif-containing proteins in phosphonate and phosphinate metabolism associated with glycolysis and phosphorylation of proteins, lipids, and carbohydrates (Figure 3B). Ranking of genes encoding GIP-9-like motif-containing proteins by fold enrichment showed the overrepresentation of KEGG pathways involved in metabolism of vitamin B6, which, in turn, is associated with metabolism of amino acids and their derivatives essential for cell growth (Figure 3D). Therefore, these metabolic pathways are essential for the functioning of the retrieved proteins.

### 2.5. Metabolic Pathways

**Figure 4:**
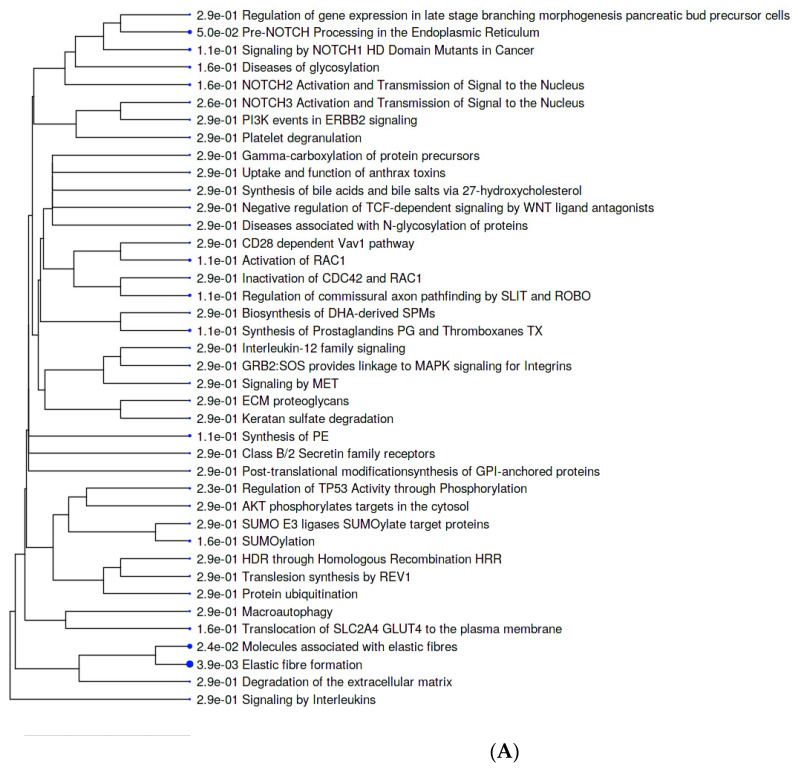

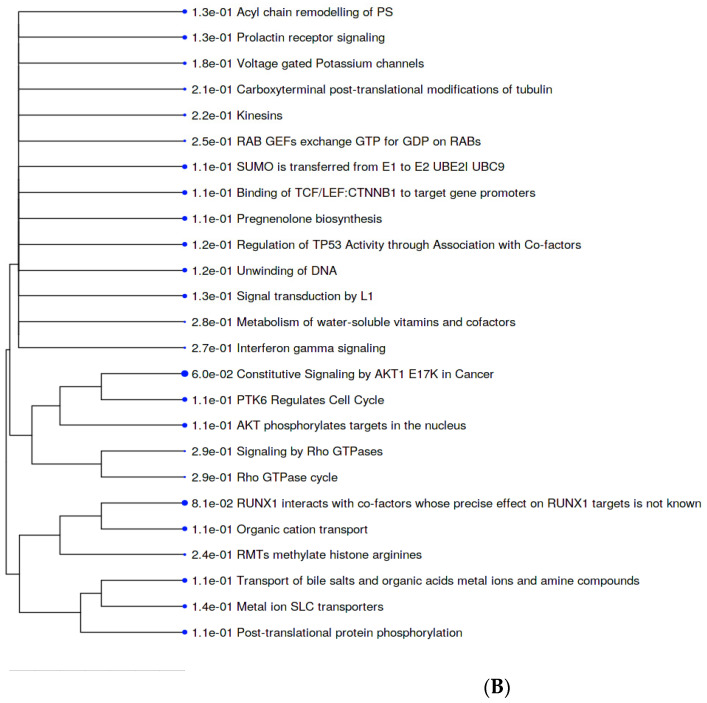
Hierarchical tree representation of the reactome metabolic pathway categories of genes encoding (**A**) AFP_14–20_-like motif-containing proteins and (**B**) GIP-9-like motif-containing proteins. The tree summarizes the correlation among significant pathways in the gene enrichment list. Pathways with many shared genes are clustered together. Larger dots indicate more significant *p*-values.

Further, we used the Reactome resource to obtain more detailed information on the involvement of the retrieved proteins in cell metabolism and signaling. Figure 4A shows that terms associated with elastic fiber formation are overrepresented among pathways that involve AFP_14–20_-like motif containing proteins. The elastic fiber proteins, such as fibulin family members, including finulin-4 (identified here), play key roles in the assembly of elastic fibers, as well as sequestering and binding of growth factors to ECM, and contain the RGD tripeptide to interact with integrins. Additionally, pathways implicated in pre-NOTCH transcription, translation, and processing were overrepresented in our work. Remarkably, nascent NOTCH peptides are cotranslationally targeted to the endoplasmic reticulum for processing and further modification in the Golgi apparatus, as well as trafficking to the plasma membrane. In addition, we found that biosynthesis of prostaglandins (PGs) and thromboxanes (TXs), synthesis of phosphatidylethanolamine (PE), activation of RAC1, diseases associated with O-glycosylation of proteins, and NOTCH1 signaling in cancer were among the significant pathways.

The most significant pathways that involve GIP-9-like motif-containing proteins included constitutive signaling mediated by Akt1 carrying the E17K mutation, which is implicated in cancer (Figure 4B). A low-frequency point mutation, E17K, in Akt1 enables binding to phosphatidylinositol-2-phosphate (PIP2) for phosphorylation by TORC2 complex, as well as activation. FOXO transcription-factor-mediated transcription of cell-cycle genes, including cyclin-dependent kinase inhibitor CDKN1A (p21Cip1), were also found among significant pathways. High significance of the runt-related transcription factor family (RUNXs), including RUNX1, RUNX2, and RUNX3, which are involved in developmental processes, immune response, and cancer, was also identified. Another pathway involved in cell-cycle regulation involves protein tyrosine kinase 6 (PTK6), which promotes cell-cycle progression by phosphorylation/inactivation of CDKN1A. Additionally, genes regulated by beta-catenin and TCF/LEF that participate in cell proliferation, differentiation, embryonic development, and tissue homeostasis were identified with high significance. Regulation of tumor suppressor gene TP53 through the association with cofactors was also identified among metabolic pathways, which involve GIP-9-like motif-containing proteins. Additionally, significant metabolic pathways included acyl chain remodeling of phosphatidylserine (PS), pregnenolone biosynthesis, and pathways that involve organic cation and metal ion solute carrier (SLC) transporters.

### 2.6. PPI Networks

**Figure 5:**
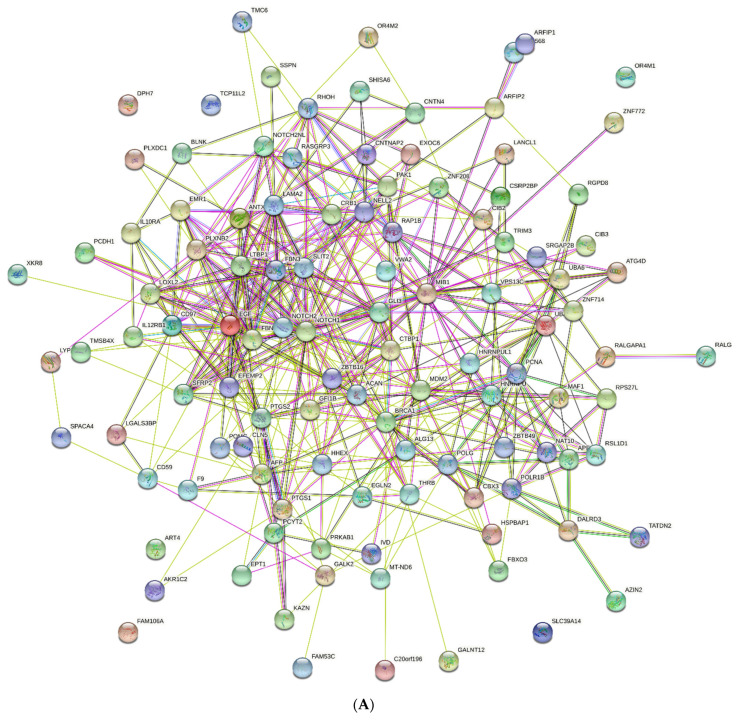

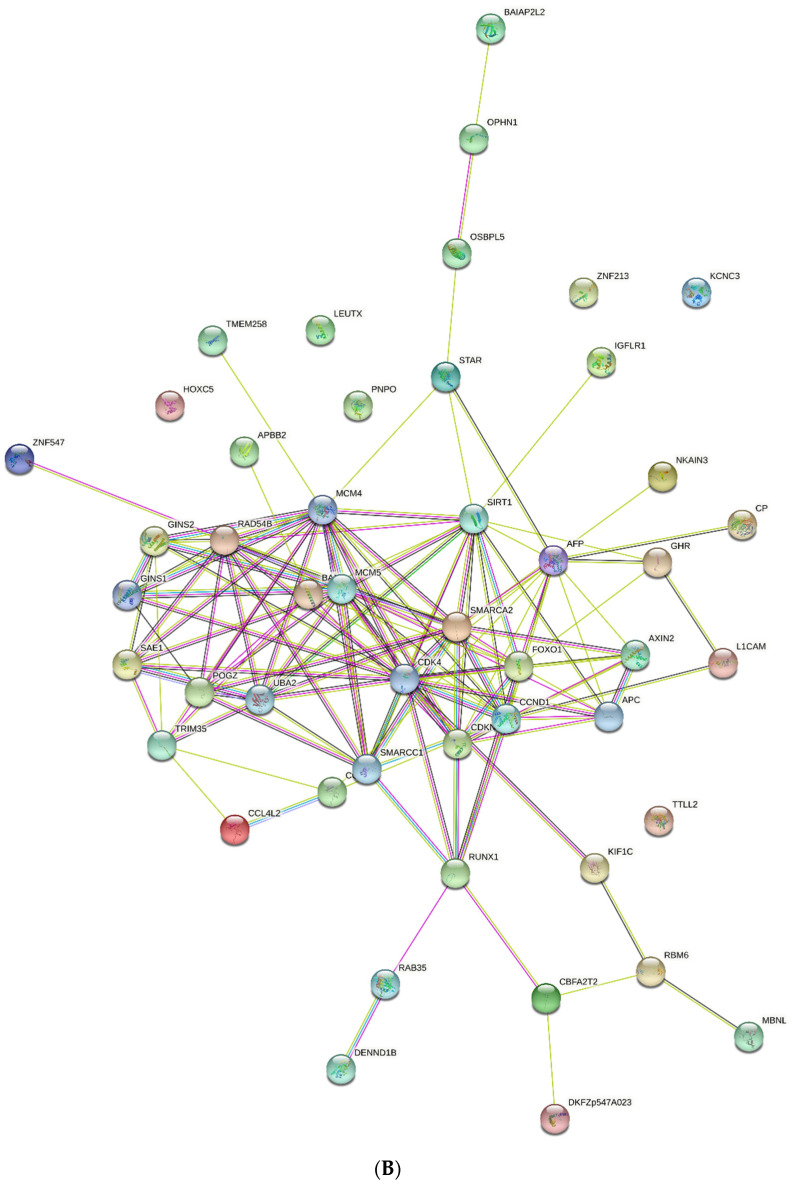
Protein–protein interaction networks constructed by STRING resource for genes encoding (**A**) AFP_14–20_-like motif-containing proteins and (**B**) GIP-9-like motif-containing proteins. ENSEMBL gene IDs or STRING-db protein IDs were used. Colored nodes—query proteins and first shell of interactions; white nodes—second shell of interactions; filled nodes—proteins of known or predicted 3D structure; empty nodes—proteins of unknown 3D structure. Known interactions: blue—from curated databases and violet (experimentally determined). Predicted interactions: red—gene fusions; green—gene neighborhood; purple—gene co-occurrence. Other interactions: lilac—protein homology; black—gene coexpression; light green—text mining.

Because SLiMs are involved in the interactions underlying protein functioning, we further undertook STRING network analysis. As shown on Figure 5A, proteins containing AFP_14–20_-like motifs constructed a PPI network with 112 nodes and 449 edges (interactions) at a confidence score of 0.150, an average node degree of 8.02, and a *p*-value of 1.6 × 10^-2^. This approach allowed for the identification of hub genes with the most interaction partners, which include *NOTCH1* and *NOTCH2* isoforms, as well as *EGF*, *FBN3*, *SLIT2*, *LTBP1*, *LAMA2*, *SFRP2*, *EMR1* (*ADGRE1*), *MIB1*, *POLR1B*, *LOXL2*, *GLI3*, *PCNA*, *CRB1*, and *PTGS2*. However, there were genes with no interactions, including *TCP11L2*, *OR4M1*, *SLC39A14*, *FAM10A*, and *ART4*. Interestingly, novel genes that were not retrieved by local alignment algorithms were identified in our PPI network. They included *NAT10*-encoding N-acetyltransferase 10, *CBX3*-encoding DNA binding chromobox protein homolog 3, *GALNT12*-encoding N-acetylgalactosaminyltransferase 12, *MT-ND6*-encoding electron transportation chain protein NADH-ubiquinone oxidoreductase chain 6, and *DPH7*-encoding diphthamide biosynthesis 7, which is essential for posttranslational modification of elongation factor 2.

The proteins that contain GIP-9-like motifs were identified to create a PPI network with 48 nodes and 133 interactions at a confidence score of 0.150 and an enrichment *p*-value of 4.26 × 10^−4^ (Figure 5B). *POGZ*, *GINS1*, *GINS2*, *MCM4*, *MCM5*, *CDK4*, *CCND1*, *SIRT1*, and *RAD548* demonstrated the hub gene properties. Additionally, there were novel genes, such as *SIRT1*-encoding NAD-dependent deacetylase sirtuin-1; *APC*-encoding adenomatous polyposis coli protein, which is a negative regulator of beta-catenin involved in Wnt signaling; and *RAB35*-encoding Ras GTPase-related protein Rab-35, which is involved in endosomal trafficking. Furthermore, two novel genes encoding DNA replication licensing factors *MCM4* and *MCM5*, which interact with *GINS2* and its isoform, *GINS1* complexes, were identified as hub genes. Cell-cycle regulator genes that interact with the retrieved *CDKN1B*, such as *CCND1*-encoding cyclin 1, as well as *CDK1*- and *CDK4*-encoding cyclin-dependent kinases 2 and 4, were among the novel genes. Additionally, the *UBA2* isoform of *UBA6* aligned to the AFP_14–20_ segment but not the GIP-9 segment, was among new genes not retrieved by local alignment.

## 3. Discussion

AFP is a major mammalian embryo-specific and cancer-related protein [17]. We previously constructed a 3D structure of human AFP [61] and performed mapping of its short linear sequences with putative and experimentally confirmed biological activities [62]. Two human AFP-derived peptides, AFP_14–20_ and GIP-9, have been chemically synthesized and experimentally studied [20,21]. Here, we undertook a search for AFP_14–20_-like and GIP-9-like SLiMs in human proteins, as well as GO-term-based comprehensive analysis of the retrieved proteins that contain both types of SLiMs of interest. The analyses were performed by categorization of the identified proteins in biological process, molecular functions, metabolic pathways, KEGG pathways, and PPI network terms. We identified both types of SLiMs in a variety of unrelated and non-homologous proteins that are involved in embryonic development and cancer progression. Surprisingly, we found that both SLiM types in multiple oxidoreductase enzymes were implicated in the regulation of redox homeostasis. Below, we discuss the implication of the most representative proteins retrieved in our work in the abovementioned cellular processes.

### 3.1. AFP_14–20_-like Motif-Containing Proteins

The majority of proteins aligned to the AFP_14–20_ segment belonged to transcription factors (Table 1). Among them was TRIM proteins, which have three types of domains at their *N*-terminus: RING finger domain, B-box zinc finger domain, and coiled-coil region. These domains provide the involvement of TRIM proteins transcriptional regulation, cytoskeletal organization, epithelial development, cell adhesion, and immune response [22]. Another retrieved transcription factor, HHEX, is involved in cell growth and differentiation, hepatic and pancreatic development, and anterior–posterior pattern specification via the Wnt signaling pathway [23]. Additionally, HHEX has been associated with type 2 diabetes, whereas zinc finger proteins are linked to the progression of various cancers [63,64,65].

NOTCH family proteins function as receptors for membrane-bound ligands Jagged-1, Jagged-2, and Delta-1 to regulate cell fate and development through the formation of transcriptional regulator complexes [24,25]. Aberrant NOTCH expression has been linked to the progression of various types of cancer [66]. Additionally, various EGF-like repeat-containing proteins, such as fibulin-2, implicated in embryonic development and tissue homeostasis [26,67] were aligned to the AFP_14–20_ segment. SRGAP2 protein, which is implicated in spatially and temporally balanced development of excitatory and inhibitory synapses [27], was also aligned to the AFP_14–20_ segment. Calcium and integrin-binding family member 2 (CIB2), which blocks translocation of sphingosine kinase 1 (SK1) to the plasma membrane, was also identified. This protein inhibits cell signaling for sensitization to TNFα-induced apoptosis and inhibition of Ras-induced neoplastic transformation [28].

F-box motif proteins, which constitute the SCF-E3 ubiquitin ligase complex of the ubiquitin-proteasome (UPS) protein degradation pathway, were also retrieved. The proteins that are degraded with this complex include translational regulatory and cell-cycle proteins during embryogenesis [29]. Ubiquitin-like modifier-activating enzyme 6 (UBA6), which activates ubiquitin and uses ubiquitin-conjugating enzyme (E2) to target proteins to proteasomal degradation, was also aligned [30]. UBA6 activates human leukocyte antigen F-adjacent transcript 10 (FAT10), which serves as 26S proteasome-targeting signal, to be involved in epithelial-mesenchymal transition (EMT), invasion, and apoptosis in hepatocellular carcinoma [31]. Dysregulation of a protein ubiquitination cascade is implicated in various human diseases, including neurodegenerative disorders and cancer [68].

Multiple proteins involved in the regulation of cell cycle, cell fusion, and apoptosis were also aligned to the AFP_14–20_ segment [32,33]. An example is ethanolamine-phosphate cytidylyltransferase, which is involved in the biosynthesis of membrane phospholipid, PE [34]. The Atg 4 cysteine proteases that are required for conjugation of Atg 8 to PE on autophagosomal membranes, a key step in autophagosome biogenesis during the macroautophagic process, were also retrieved [35]. Interestingly, AFP_14–20_-like motifs were found in CTP:PE cytidylyltransferase, which is involved in phospho-ethanolamine biosynthesis from ethanolamine [36]. Upregulated phosphoethanolamine biosynthesis is required to meet increased demands in energy and metabolites for T-cell activation, cellular proliferation, and cancer cell adaptation [69]. Immune response regulators aligned to AFP_14–20_ include B-cell linker protein, which is crucial for B-cell differentiation. Downregulation/mutation in the *BLNK* gene has been shown to induce acute lymphoblastic leukemia through JAK3 signaling [37].

Living organisms have adapted to oxidative stress conditions via reversible post-translational chemical modifications of redox-sensitive amino acid residues in intracellular effectors of signal transduction pathways (protein kinases and protein phosphatases), transcription factors, etc. [70]. Dysregulation of these mechanisms has been associated with various human diseases, including cancer. Proteins involved in redox regulation include 3-alpha hydroxysteroid dehydrogenase III, which belongs to steroidogenic oxidoreductase enzymes and uses NADPH or NADH, cofactors involved in ROS generation [38]. The retrieved proteins include dual cyclooxygenase and peroxidase, as well as prostaglandin G/H synthase 1, which is involved in the biosynthesis of prostanoids and ROS generation [39]. Additionally, glutathione S-transferase LANCL1 is involved in oxidative stress response and is overexpressed in prostate cancer cells [40]. LANCL1 causes the expression of glucose transporters, as well as mitochondrial uncoupling and respiration via the AMPK/PGC-1α/Sirt1 pathway [71]. HSPB-associated protein 1 (HSPBAP1) exhibits oxidoreductase activity, and its overexpression has been observed in prostate cancer samples [41]. Another oxidoreductase, prolyl hydroxylase, catalyzes hydroxylation of proline residues in hypoxia-inducible factor-1α (HIF-1α) and other target proteins, such as ATF4, IKBKB, and CEP192 under hypoxia conditions [42]. This leads to pVHL (von Hippel–Lindau protein)-dependent ubiquitination and rapid proteasomal degradation of HIF-1α, which is implicated in cancer progression [72].

### 3.2. GIP-9-like Motif-Containing Proteins

Proteins aligned with high significance to the GIP-9 segment of human AFP include various isoforms of C-C motif chemokine 4-like (Table 2), which has been shown to promote human trophoblast migration at the fetoplacental interface during embryonic development [43]. Additionally, other development-associated proteins, including Wnt-signaling regulators, such as AXIN2, were retrieved from the UniprotKB database. Wnt signaling is involved in embryonic pattern formation and tissue morphogenesis, whereas dysregulation of Wnt signaling has been implicated in various cancer types, including colorectal and hepatocellular carcinoma [44]. Additionally, cell adhesion proteins were identified, such as L1 cell adhesion molecule (L1CAM or CD171), a transmembrane protein and member of the immunoglobulin superfamily, which plays a major role in nervous system development, as well as cancer cell migration and invasion [45].

Transcription factor, the paired (PRD)-like leucine twenty homeobox (LEUTX) domain protein, is expressed almost exclusively in human embryos during preimplantation development [46]. HOXC5 transcription factor is also involved embryonic development; however, the deregulation of HOXC5 has been shown to contribute to activation of the *TERT* gene in human cancers [47]. Another transcription factor, FoxO1, is overexpressed and became acetylated due to the dissociation from histone deacetylase sirtuin-2 (SIRT2) in response to oxidative stress. This causes its binding to Atg7, the E1-like protein, leading to cancer cell death via autophagy and tumor suppression [48,49]. Additionally, RUNX family transcriptional regulators, key regulators of normal embryonic development overexpressed in cancer, were among the retrieved proteins [50].

Among cell-cycle regulators, BTG3-associated nuclear protein and cyclin-dependent kinase inhibitor 1B are involved in the cell-cycle G_1_/S transition, playing tumor-suppressor roles [51]. Additionally, various proteins involved in DNA replication, repair, and recombination were retrieved from the UniprotKB knowledgebase, identified as containing GIP-9-like motifs. An example is DNA replication complex GINS protein, a part of the human replisome, a molecular machine responsible for accurate chromosome replication [52].

Proteins with receptor activity include IGF-like family receptor 1, which is implicated in T-cell-mediated inflammation and associated with the prognosis of various cancers correlating with immune cell infiltration [53,54]. Another example is brain-specific angiogenesis inhibitor (BAI)-associated protein 2-like 1 (BAIAP2L1), known as insulin receptor tyrosine kinase (RTK) substrate. It belongs to putative G-protein-coupled receptors, with a wide spectrum of cellular activities, including inflammation and tumorigenesis [55,56]. E3 ubiquitin-protein ligase TRIM35, which participates in multiple biological processes, including cell death, glucose metabolism, and innate immune response to viral infection, was also found to contain the GIP-9-like motif [57].

Proteins involved in redox regulation include ceruloplasmin, an enzyme with ferroxidase activity and a major copper-binding protein in the blood, which plays a key role in redox homeostasis and metabolic regulation [58]. Additionally, PNPO, which converts pyridoxine 5’-phosphate into pyridoxal 5’-phosphate (PLP), an active form of vitamin B6, is implicated in several types of cancer and was aligned to GIP-9 [59]. Another example is a growth hormone receptor (GHR) involved in metabolic regulation; its deficiency causes upregulation of enzymes involved in amino acid catabolism, urea cycle, and tricarboxylic acid cycle, as well as reduced mitochondrial import of fatty acids for beta-oxidation [60].

## 4. Materials and Methods

### 4.1. Search for Short Linear Motifs

The FastA suite of the European Molecular Biology Laboratory-European Bioinformatics Institute (EMBL-EBI) was applied (https://www.ebi.ac.uk/Tools/sss/fasta/ (accessed on 22 November 2021)) [73] for local sequence alignment. Two human AFP-derived short sequences, LDSYQCT and EMTPVNPGV, were used as query sequences. The search was performed against the UniprotKB human taxonomic subset [74]. GLSEARCH (version 36.3.8 h) algorithm provided the most optimal search to match the query sequences. The BLOSUM50 matrix and the following parameters were used to obtain as many as 500 alignments: gap open: 10; gap extension: 2; KTUP: 2; expectation value (E-value) upper unit: 10 and lower unit: 0.

### 4.2. Gene Ontology Analysis

Lists of genes encoding the retrieved human proteins were composed for further analysis. Gene ontology resource (http://geneontology.org/ (accessed on 15 December 2021)) was utilized for gene enrichment analysis in biological process categories. The gene list analysis option of PANTHER17.0 classification system (http://pantherdb.org/ (accessed on 23 December 2021)) was used for this purpose [75]. GO-Slim annotation and a statistical overrepresentation test were applied. Additionally, the GeneCards human gene database (https://www.genecards.org/ (accessed on 20 January 2022)) annotations were applied for gene categorization [76]. All query genes were retrieved from the UniprotKB knowledgebase and then converted to ENSEMBL [77] gene IDs.

### 4.3. Gene Set Enrichment Analysis

The ShinyGO v0.75 suite (http://bioinformatics.sdstate.edu/go/ (accessed on 8 February 2022)) was utilized [78] for further detailed gene set enrichment analysis on the basis of molecular functions and metabolic pathway categories. Both fold enrichment and gene enrichment options were applied with a color heatmap of −log10(FDR). Fold enrichment is calculated by the percentage of genes in the list belonging to a pathway divided by the corresponding percentage of genes in the background, i.e., the whole human genome. Characteristics of genes in our lists were compared to those of genes of the whole human genome, and Student’s t-test was applied. Lolipop chats with an aspect ratio of 1.5 were utilized for visualization. FDR was calculated based on nominal *p*-value from the hypergeometric test in order to determine the likelihood of enrichment by chance.

### 4.4. KEGG Pathway Enrichment Analysis

The KEGG pathway database [79] analysis option of the ShinyGO v0.75 suite was used with an FDR cutoff of 0.4 for both gene enrichment and fold enrichment versions. Bar plot charts with an aspect ratio of 1.5 were used for visualization to generate log10 (FDR) heat maps for each category.

### 4.5. Metabolic Pathway Analysis

The reactome pathway database (https://reactome.org/ (10 March 2022)) was applied with the gene list analysis option and functional annotation report [80]. Additionally, the Curated.Reactome option of the ShinyGO v0.75 suite was used to assess metabolic pathway enrichment analysis. A *p*-value (FDR) of 0.4 was used to identify as many as 40 pathways. The minimum pathway size was 5, and the maximum pathway size was 2000.

### 4.6. PPI Network Analysis

The STRING (https://string-db.org/ (accessed on 17 March 2022)) suite was used for PPI network enrichment analysis [81]. Full STRING network type, a confidence score of 0.150, and an FDR stringency of 1.0 percent were applied.

## 5. Conclusions

Short linear motifs with sequence similarity to two biologically active sites of human AFP were identified in multiple non-homologous and unrelated proteins with the use of a local alignment algorithm. Gene ontology term-based categorization was performed on the proteins retrieved from the UniprotKB database. Gene set enrichment analysis in biological process, molecular functions, metabolic pathways, KEGG pathways, and PPI network categories allowed for identification of functional classes of the retrieved proteins. Transcription factors, proteins involved in DNA replication/repair, cell-cycle progression, signal transduction, ubiquitin-mediated protein degradation, immune response, and oxidoreductase enzymes were aligned to both types of SLiMs. The majority of proteins were involved in embryonic development, cancer, and redox regulation. Our data support the concept that proteins are composed of evolutionarily conserved short linear segments that are incorporated into their primary structure as functional building blocks to be reused in a variety of non-homologous proteins.

## Figures and Tables

**Table 1:** Selected human proteins retrieved from UniprotKB knowledgebase containing AFP_14–20_-like motifs.

Protein Name	Entry Code	Gene Symbol	Alignment	Aa Positions	Identity	E-Value	GO Molecular Functions	GO Biological Processes	Reference
Tripartite motif-containing protein 3 (RING finger protein HAC1)	TR:Q1KXY7	*TRIM3*	LDSYQCT : : . : : : LDRYQCP	26–32	71.4%	2.2 × 10^−4^	Metal ion binding, ubiquitin-protein ligase/transferase activity	Transcriptional regulation, UPS-mediated protein degradation	[22]
Zinc finger protein 714	TR:A0A087WV13	*ZNF714*	LDSYQCT . : : : : . ENSYQCE	15–21	57.1%	3.0 × 10^−2^	Transcription factor	Transcriptional regulation	[22]
Hematopoietically-expressed homeobox protein HHEX	TR:F8VU08	*HHEX*	LDSYQCT : : : : : . LDSSQCS	59–65	71.4%	9.4 × 10^−4^	DNA binding, transcription activator activity	Transcriptional regulation, anterior–posterior pattern specification, B- cell differentiation	[23]
Neurogenic locus notchhomolog protein 2	TR:A0A494C1F1	*NOTCH2*	LDSYQCT : . : . : : RDTYECT	87–93	57.1%	7.9 × 10^−2^	Calcium ion binding, signaling receptor activity	Tissue morphogenesis, cell fate determination	[24]
von Willebrand factor A domain-containing protein 2	SP: Q5GFL6-2	*VWA2*	LDSYQCT : : : : : LDGYQCL	315–321	71.4%	0.36	Calcium binding activity	Cell–matrix adhesin, insulin-receptor signaling	[25]
EGF-containing fibulin-like extracellular matrix protein 2	TR: E9PRQ8	*EFEMP2*	LDSYQCT . : : : : : PGSYQCT	144–150	71.4%	4.0	Calcium ion binding	Extracellular matrix assembly, developmental processes	[26]
Slit-Robo RhoGTPase-activating protein 2B	TR:A0A087 × 1G6	*SRGAP2B*	LDSYQCT : : . : : . LYSHQCS	22–28	57.1%	0.17	GTPase activator activity	Neuronal morphogenesis developmental process	[27]
Calcium and integrin-binding family member 2	TR:H0YND4	*CIB2*	LDSYQ-CT : : . : : : : LDNYQDCT	13–20	75.0%	3.3 × 10^−2^	Calcium ion binding, integrin binding	Calcium ion homeostasis, response to ATP	[28]
F-box protein Fbx3	TR: Q9UKC5	*FBX3*	LDSYQCT : : . : . : . LDDYRCS	137–143	57.1%	4.0 × 10^−2^	Ubiquitin-protein transferase activity	Protein ubiquitination and degradation	[29]
Ubiquitin-like modifier-activating enzyme 6	TR:Q2MD40_	*UBA6*	LDSYQCT : : . : : : LDKYQCV	151–157	71.4%	5.0 × 10^−3^	ATP binding, ubiquitin-activating enzyme activity	Response to DNA damage, protein ubiquitination, embryonic development	[30,31]
Epidermal growth factor	TR:Q6QBS2	*EGF*	LDSYQCT : : . : : . LDKYACN	26–32	57.1%	3.8 × 10^−2^	Growth factor activity	Cell proliferation and survival	[32]
Proliferating cell nuclear antigen	TR:Q7Z6A3	*PCNA*	LDSYQCT . : . : . : FDTYRCD	57–63	42.9%	0.42	DNA binding	Cell cycle regulation, DNA replication and repair	[33]
Ethanolamine-phosphate cytidylyltransferase	TR:I3L1F9	*PCYT2*	LDSYQCT : : . : . : . LDKYNCD	24–30	57.1%	5.7 × 10^−3^	Catalytic activity	Biosynthetic process, cell division, cell fusion, and apoptosis	[34]
Cysteine protease ATG4D	SP: Q86TL0-2	*ATG4D*	LDSYQCT : . : . . : : LESFHCT	40–46	57.1%	0.18	Peptidase activity	Apoptosis/autophagy/mitophagy/proteolysis	[35]
CTP:phosphoethanolamine cytidylyltransferase	TR:I3L1C4	*PCYT2*	LDSYQCT : : . : . : LDKYNCD	24–30	57.1%	0.21	Transferase activity	Biosynthetic process	[36]
B-cell linker protein	TR: Q2MD40	*BLNK*	LDSYQCT . : : : . : MDSYSCL	1–7	57.1%	9.7 × 10^−4^	SH2-domain binding, signaling adaptor activity	B-cell differentiation, immune and inflammatory response	[37]
3-alpha hydroxysteroid dehydrogenase III	TR:Q1KXY7	*AKR1C2*	LDS – YQCT . : : : : : MDSKYQCV	1–7	62.5%	4.4 × 10^−5^	Oxidoreductase, metabolic activity	Steroid hormone metabolism	[38]
Prostaglandin G/H synthase 1	SP: P23219-3	*PTGS1*	LDSYQCT : : . : : : LDRYQCD	26–32	71.4%	0.35	Cyclooxygenase/peroxidase activity, heme binding, metal ion binding	Response to oxidative stress, inflammatory process	[39]
Glutathione S-transferase LANCL1	TR:H7C2E3	*LANCL1*	LDSYQCT : : : : CDAYQCA	59–65	57.1%	0.22	Glutathione binding, zinc ion binding	Oxidative stress response	[40]
HSPB1-associated protein 1	SP: Q96EW2-2	*HSPBAP1*	LDSYQCT : : : : : : LDSYGCN	176–182	71.4%	0.12	Oxidoreductase, dioxygenase activity	Brain development	[41]
Prolyl hydroxylase EGLN2	TR:M0R2X9	*EGLN2*	LDSYQCT : : : . : LPSYHCP	45–51	57.1%	2.5	Dioxygenase activity, oxygen sensor activity	Cell redox homeostasis, response to hypoxia	[42]

Note: colons between the aligned sequences indicate identity of the residues, whereas dots indicate similarity between residues.

**Table 2:** Selected human proteins retrieved from UniprotKB knowledgebase containing GIP-9-like motifs.

Protein Name	Entry Code	Gene Symbol	Alignment	Aa Positions	Identity	E-Value	Go Molecular Functions	Go Biological Processes	Reference
Zinc finger protein 547	TR: M0QYW2	*ZNF547*	EMTPVNPGV : : . . : : : EEAPLEPGV	60–68	55.6%	3.4	DNA binding, metal ion binding, transcription factor activity	Transcriptional regulation	[22]
C-C motif chemokine 4-like	SP: Q8NHW4-7	*CCL4L1*	EMTPVNPGV . : : : . : : ALTPVSPGS	31–39	66.7%	1.7 × 10^−2^	Chemokine activity	Response to INF-γ, IL-1, and TNF-α; cell signaling	[43]
Axin 2	TR: A0A024R8M3	*AXIN2*	EMTPVNPGV : : : : : . : . EMTPVEPAT	361–369	66.7%	1.7	Beta-catenin binding, ubiquitin protein ligase binding	Regulation of Wnt signaling, cell death, bone mineralization	[44]
L1 cell adhesion molecule	TR: Q7Z2J6	*L1CAM*	EMTPVNPGV . : . : : . : ATSP I NPAV	54–62	44.4%1	1.0	Cell adhesion molecule activity	Nervous system development	[45]
Paired-like homeodomain transcription factor LEUTX	SP: A8MZ59-1	*LEUTX*	EMTPVNPGV . . . : : . : : . N I RPVSPG I	69–77	44.4%	2.3	DNA binding activity	Transcriptional regulation, embryogenesis	[46]
Homeobox protein Hox-C5	SP: Q00444	*HOXC5*	EMTPVNPGV : . : . : : : . EAAPLNPGM	90–98	55.6%	0.48	DNA-binding activity, transcription factor	Anterior/posterior specification, embryonic development	[47]
Forkhead box protein O1	SP: Q12778	*FOXO1*	EMTPVNPGV : : : : . : : : IMTPVDPGV	476–484	77.8%	0.12	DNA-binding activity, transcription factor	Transcriptional regulation, metabolic response to oxidative stress	[48,49]
RUNX1/CBFA2T2 fusion protein type 1	TR:D1LYX4	*RUNX1/CBFA2T2*	EMTPVNPGV . : . : : : . PLP P I NPGG	50–58	44.4%	8.5	Transcription corepressor activity	Transcriptional regulation	[50]
Cyclin-dependent kinase inhibitor 1B	TR: H7C2T1	*CDKN1B*	EMTPVNPGV : : : . : : . EQTPKKPGL	91–99	55.6%	2.3	Cyclin binding, chaperone binding	Cell-cycle regulation, autophagy, response to chemicals	[51]
DNA replication complex GINS protein PSF2	SP: Q9Y248	*GINS2*	EMTPVNPGV . . : . : : : . DLGPFNPGL	32–40	44.4%	7.2	DNA binding	DNA replication, DNA repair	[52]
IGF-like family receptor 1	TR: K7ESC2	*IGFLR1*	EMTPVNPGV . : : . : : : . PLTPGNPGA	124–132	55.6%	2.7	Receptor activity	IGF-mediated signaling, inflammation process	[53,54]
Brain-specific angiogenesis inhibitor 1-associated protein 2-like protein 2	TR: B0QYF0	*BAIAP2L2*	EMTPVNPGV : : : . : : : PMTPMNPGN	98–106	66.7%	4.5 × 10^−2^	Cadherin-binding and cytoskeletal-binding activities	Actin cytoskeleton organization, brain development	[55,56]
E3 ubiquitin-protein ligase TRIM35	TR: H0YBF3	*TRIM35*	EMTPVNPGV : . . : : . : : . EPEPVQPGM	33–41	55.6%	4.6	Zinc ion binding, ubiquitin-protein ligase activity	Protein ubiquitination, innate immune response, apoptotic process	[57]
Ceruloplasmin	TR: H7C5N5	*CP*	EMTPVNPGV : : : . : . EMFPRTGG I	162–170	55.6%	2.8	Oxidoreductase activity, copper binding	Redox homeostasis	[58]
Pyridoxine-5’-phosphate oxidase	TR: A0A286YF38	*PNPO*	EMTPVNPGV : . : . : : EVPPLGPGL	46–54	44.4%	1.5	Oxidoreductase activity	Biosynthetic process	[59]
Growth hormone receptor	TR: Q9NRZ8	*GHR*	EMTPVNPGV . . . : : : : . SLQSVNPGL	11–19	44.4%	1.3	Cytokine receptor activity	Response to stimulus, cell signaling	[60]

Note: colons between the aligned sequences indicate identity of the residues, whereas dots indicate similarity between residues.

## Data Availability

Data are available in a publicly accessible repository. The data presented in this study are openly available in FigShare at doi:10.6084/m9.figshare.19806037.
